# Deep learning-based Alzheimer's disease detection: reproducibility and the effect of modeling choices

**DOI:** 10.3389/fncom.2024.1360095

**Published:** 2024-09-20

**Authors:** Rosanna Turrisi, Alessandro Verri, Annalisa Barla

**Affiliations:** ^1^Department of Informatics, Bioengineering, Robotics and System Engineering (DIBRIS), University of Genoa, Genoa, Italy; ^2^Machine Learning Genoa (MaLGa) Center, University of Genoa, Genoa, Italy

**Keywords:** deep learning, Alzheimer's disease, data augmentation, model depth, reproducibility

## Abstract

**Introduction:**

Machine Learning (ML) has emerged as a promising approach in healthcare, outperforming traditional statistical techniques. However, to establish ML as a reliable tool in clinical practice, adherence to best practices in *data handling*, and *modeling design and assessment* is crucial. In this work, we summarize and strictly adhere to such practices to ensure reproducible and reliable ML. Specifically, we focus on Alzheimer's Disease (AD) detection, a challenging problem in healthcare. Additionally, we investigate the impact of modeling choices, including different data augmentation techniques and model complexity, on overall performance.

**Methods:**

We utilize Magnetic Resonance Imaging (MRI) data from the ADNI corpus to address a binary classification problem using 3D Convolutional Neural Networks (CNNs). Data processing and modeling are specifically tailored to address data scarcity and minimize computational overhead. Within this framework, we train 15 predictive models, considering three different data augmentation strategies and five distinct 3D CNN architectures with varying convolutional layers counts. The augmentation strategies involve affine transformations, such as *zoom, shift*, and *rotation*, applied either concurrently or separately.

**Results:**

The combined effect of data augmentation and model complexity results in up to 10% variation in prediction accuracy. Notably, when affine transformation are applied separately, the model achieves higher accuracy, regardless the chosen architecture. Across all strategies, the model accuracy exhibits a concave behavior as the number of convolutional layers increases, peaking at an intermediate value. The best model reaches excellent performance both on the internal and additional external testing set.

**Discussions:**

Our work underscores the critical importance of adhering to rigorous experimental practices in the field of ML applied to healthcare. The results clearly demonstrate how data augmentation and model depth—often overlooked factors– can dramatically impact final performance if not thoroughly investigated. This highlights both the necessity of exploring neglected modeling aspects and the need to comprehensively report all modeling choices to ensure reproducibility and facilitate meaningful comparisons across studies.

## 1 Introduction

Advanced Machine Learning (ML) techniques have proven to be highly effective in healthcare applications, such as cancer detection and prognosis (Cruz and Wishart, 2006; Sajda, 2006; Kourou et al., 2015; Shen et al., 2019; Chaunzwa et al., 2021), heart diseases prediction (Mohan et al., 2019; Palaniappan and Awang, 2008), and neurodegenerative diseases' diagnosis (Pereira et al., 2016; Montolío et al., 2021). However, it is still premature to assert that ML is ready to be employed as a standard in clinical practice. For instance, in Roberts et al. (2021), the authors reviewed thousands of papers on the use of ML to detect COVID-19 and found that none achieved the robustness and reproducibility required for medical use. This issue is not specific to ML methods for COVID-19 detection but involves the entire ML community (Ioannidis, 2005; Pineau et al., 2021), particularly the field of ML in healthcare (Stupple et al., 2019; Beam et al., 2020; Heil et al., 2021). To address this issue, Luo et al. (2016) asked 11 researchers with expertise in biomedical ML to produce a set of rules ensuring that ML models within clinical settings are sufficiently reported. These rules mainly relate to paper writing, providing a checklist for each article section. Although Luo et al. (2016) offers a useful tool for checking final manuscripts, it does not identify specific practices for developing ML methods in healthcare and is often very general when it comes to report ML model details (e.g., identifying if the study is retrospective/prospective and if the prediction task is regression/classification).

In our manuscript, we identify an essential set of practical guidelines, and we highlight the importance of fully adhering to them. To demonstrate this, we present a practical application of ML in healthcare by following these guidelines and demonstrating the impact of modeling choices on the final performance. Specifically, we focus on Deep Learning (DL) for Alzheimer's Disease (AD) diagnosis. AD is the most common type of dementia, impacting over 30 million individuals globally. It is characterized by (i) a pre-symptomatic stage where pathological molecular changes and neuronal dysfunctions occur at brain level, (ii) a prodromal stage identified as mild cognitive impairment (MCI) syndrome; (iii) an early-stage where cognitive symptoms of AD become more evident; (iv) a late stage with overt dementia. This progressive neurodegenerative disorder leads to cognitive and functional decline, impairing daily activities and eventually resulting in death. Hence, timely and accurate diagnosis of AD is crucial for effective treatments. Structural Magnetic Resonance Imaging (MRI) has proven to be a powerful tool for predicting AD due to its ability to visualize detailed brain structures and identify changes associated with the disease, such as hippocampal atrophy (Jack et al., 2000; Van De Pol et al., 2006), cortical thinning (Du et al., 2007), and brain volume loss (Pini et al., 2016).

In this study, we leverage low-resolution MRI scans and address the challenge of discriminating patients with AD from Cognitively Normal (CN) subjects using a 3D-Convolutional Neural Network (CNN) (LeCun et al., 1995). We combine different data augmentation strategies and CNN depths, creating a total of 15 DL models. We show that these modeling choices can lead to significant variations in prediction accuracy, up to 10%. The best model demonstrates excellent accuracy on the testing set and good properties of generalization to an external dataset. It is worth noting that the proposed approach can be readily extended to other modeling choices and healthcare applications.

The paper is structured as follows. The Materials and Methods section includes the guidelines for ML reliability and reproducibility, and introduces state-of-the-art studies in the AD field. Then, it details data handling and the experimental setup, including modeling challenges and choices made. The Results section evaluates the effect of the modeling choices, comparing augmentation strategies and architectures. The Discussion section relates findings to state-of-the-art studies and illustrates future perspectives.

## 2 Materials and methods

### 2.1 Guidelines

To begin, we summarize the general guidelines for reliable and reproducible ML pertaining to two key aspects: *data handling*, and *model design and assessment*.

**Data handling (D)**

1. Data collection/selection should align with the scientific problem at hand (e.g., utilizing cross-sectional data for diagnostic confirmation or longitudinal data for prognostic purposes), avoiding bias and information leakage (Saravanan et al., 2018).
2. Data quality should be assessed by identifying missing values and inconsistencies, and improved by applying appropriate imputation and cleaning methods (Lin and Tsai, 2020).
3. Data harmonization can be used to compensate for heterogeneous data from different acquisition techniques (Kourou et al., 2018).
4. Data augmentation can be employed as a solution for small sample size or unbalanced samples per class, a common case in the biomedical field.
5. The whole data handling process should be described in details in order to ensure reproducibility.

**Model design and assessment (M)**

1. The versioned code used for conducting the experiments should be publicly shared to ensure transparency and reproducibility.
2. Every decision in the design of the predictive model should be justified, with recognition of uncontrollable factors (Haibe-Kains et al., 2020).
3. Details about the samples used in the training/testing split should be disclosed to guarantee benchmarking.
4. A well-designed experiment should avoid assessing results on a non-representative testing set. To this aim, resampling strategies (Batista et al., 2004) such as k-fold cross-validation or boosting can be utilized to comprehensively assess the model's performance. Further, models based on random weights initialization should be repeated for different trials in order to assess their stability.
5. The performance metrics should be chosen according to the specific scientific objectives of the study (Sokolova and Lapalme, 2009; Chicco and Jurman, 2020).
6. Testing the model on external datasets is ideal to evaluate its generalization properties (Basaia et al., 2019).

These guidelines are followed throughout the rest of the paper and referenced within the text whenever a rule is applied in the experiments.

### 2.2 State of the art

AD is a neurodegenerative disease and the most common form of dementia globally, characterized by progressive neurodegeneration, leading to cognitive and functional decline, impaired daily activities, and eventually, death (Wu et al., 2017; Dubois et al., 2016). Brain imaging, particularly MRI scans, plays a crucial role in diagnosing AD by providing detailed insights into the structural brain changes associated with the disease. In recent years, ML models have shown significant potential in utilizing imaging data to improve automated AD diagnosis (Yu et al., 2022) and predict AD-related brain abnormality (Zong et al., 2024). For instance, Zuo et al. (2024) use multiple brain image modalities with an adversarial learning strategy for AD progression prediction and to identify abnormal brain connections. Similarly, Pan et al. (2024) proposes a generative adversarial network with a decoupling module to detect abnormal neural circuits.

As reported in Arya et al. (2023), the Alzheimer's Disease Neuroimaging Initiative (ADNI) dataset (Mueller et al., 2005) is the most frequently employed dataset in AD studies based on ML and DL approaches. ADNI comprises heterogeneous datasets collected during different temporal phases (ADNI1, ADNI/GO, ADNI2, and ADNI3), each characterized by varying MRI acquisition protocols. ADNI1 includes longitudinal acquisitions on 1.5T and 3T scanners with T1- and T2-weighted sequences; ADNI-GO/ADNI2 contains imaging data acquired at 3T with similar T1-weighted parameters to ADNI1; ADNI3 exclusively utilizes MRI obtained from 3T scanners. Further, within a temporal phase, multiple acquisitions are done at different time steps (e.g., baseline, screening, follow up).

The heterogeneity of ADNI allowed for many experimental setups in the literature, with varying results depending on sample size [ranging from hundreds (Liu et al., 2014; Alinsaif and Lang, 2021; Long et al., 2017; Korolev et al., 2017) to thousands (Salehi et al., 2020; Basaia et al., 2019)], images resolution, or sequence type. However, this variability and the lack of a universally recognized benchmark have hindered fair comparisons of published models. Another consequence is that AD studies are more susceptible to information leakage. In Wen et al. (2020), the authors reviewed 32 studies using CNN models for AD diagnosis and found that about 50% of them reported biased results due to data leakage. These factors underscore the essential need for carefully selecting the dataset (D1), reporting details on data processing (D5, M3), taking into account the dataset size (D4, M3, M4) and choosing the model (M2) and the evaluation metrics accordingly (M5). In the rest of the section, we discuss state of the art (SOTA) studies on MRI-based AD classification using ADNI and describe their experimental approaches in relation to the criteria D and M. We emphasize that a systematic review is behind the purpose of this work, which has the scope of highlighting good and bad practices in ML for healthcare.

We considered the studies reported in a recent PRISMA-based review (Arya et al., 2023), selecting 8 articles that used solely MRI scans from ADNI dataset (Mehmood et al., 2021; Li and Yang, 2021; Pan et al., 2020; Alickovic et al., 2020; Korolev et al., 2017; Yue et al., 2019; Xiao et al., 2017; Tong et al., 2014). To increase the sample of DL-based articles, we further considered three SOTA articles (Salehi et al., 2020; Basaia et al., 2019; Ghaffari et al., 2022), for a total of 11 articles. We found that none of them fully adhered to the guidelines listed in the previous section. In particular:

- D1: 73% of studies did not report the ADNI phase, and 91% did not specify the time step (e.g., baseline, follow-up). This information is crucial to ensure that baseline and follow-up data are not mixed, thereby preventing data leakage. Additionally, 27% of studies did not provide information about MRI resolution (i.e., 1.5T or 3T).
- D4: Data augmentation is applied in only 4 papers (Mehmood et al., 2021; Pan et al., 2020; Basaia et al., 2019; Ghaffari et al., 2022). These papers lack important details, such as transformation parameters and the size of the final training set.
- M1: Only the authors in Korolev et al. (2017) provided the code used for data processing and modeling.
- M2: Only 27% of the works considered different model architectures. Additionally, none of the DL approaches explored model depth as a hyperparameter.
- M3: Three articles split the dataset into training/testing following previous work, whereas the remaining ones did not detail the samples in the splits, preventing benchmarking.
- M4: Resampling strategies were not used in 45% of experiments. Furthermore, no DL-based methods tested model robustness to random weight initialization.
- M5: 91% of studies adopted multiple evaluation metrics. However, standard deviation for resampling strategies was reported in only three papers.
- M6: Generalization across datasets was tested and reported in only two articles.

Note that D2 and D3 are not evaluated here as data quality is ensured by ADNI experts and none of the considered studies rely on different acquisition techniques.

The literature review reveals that none of the considered SOTA studies are fully reproducible due to the absence of available validated code, insufficient details about data processing and augmentation, and lack of information about dataset splits and experimental specifics. Furthermore, the reliability of these works is sometimes limited by unrepresentative testing sets and the lack of evaluation on external datasets. It is also interesting to note that the number of employed samples varies from 170 to 1,662, with a median of 433, a mean of 653, and a standard deviation of 495. This, along with the variability in MRI resolution, makes model comparisons unfeasible. Finally, we noted that model depth and data augmentation strategy (in terms of the number of augmented samples and types of transformations) were completely neglected factors. This led us to investigate whether and to what extent these two modeling choices impact the classification task.

### 2.3 Data

**Figure 1:**
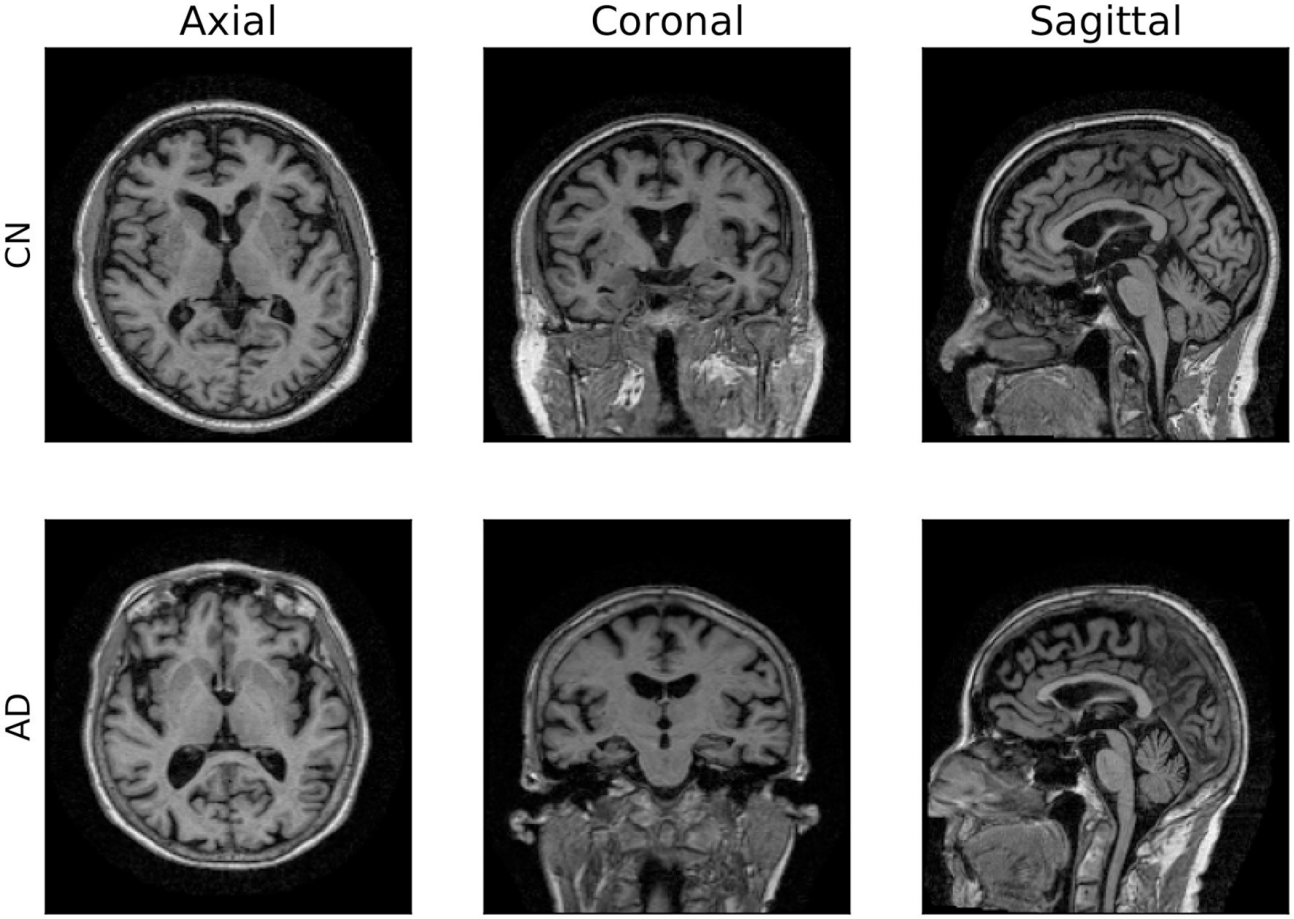
2D visualization of 3D MRI scans. Axial, coronal and sagittal planes of two brain images from ADNI dataset.

For our experiments, we adopted the ADNI dataset (Mueller et al., 2005) considering T1-weighted 1.5T MRI scans from the ADNI1 data collected during screening, which is the baseline exam. This includes 550 MRI exams from 307 CN subjects and 243 AD patients. Additionally, we used an ADNI1 subset of 80 3T MRI exams as an external testing set, to evaluate the best model in a *domain shift* setting (Buchanan et al., 2021). Table 1 reports demographic details about the two datasets (D1). We recall that MRI exams are three-dimensional data describing the structure of the brain. Figure 1 displays a 2D projection of brain images captured from a CN subject (first row) and an AD patient (second row) on the *sagittal, coronal*, and *axial* planes. All data were preprocessed by ADNI experts, ensuring data quality and harmonization (D2, D3; more information in Supplementary Section 1).

**Table 1:** ADNI1 demographic description.

**1.5T**	**CN**	**AD**
Subjects	307	243
Age	75.2 ± 7.6	75.9 ± 5.0
Sex (M/F)	159/148	130/113
**3T**	**CN**	**AD**
Subjects	47	33
Age	75.1 ± 3.9	74.0 ± 8.1
Sex (M/F)	18/29	11/22

1.5 and 3T datasets.

#### 2.3.1 Data augmentation (D4)

**Figure 2:**
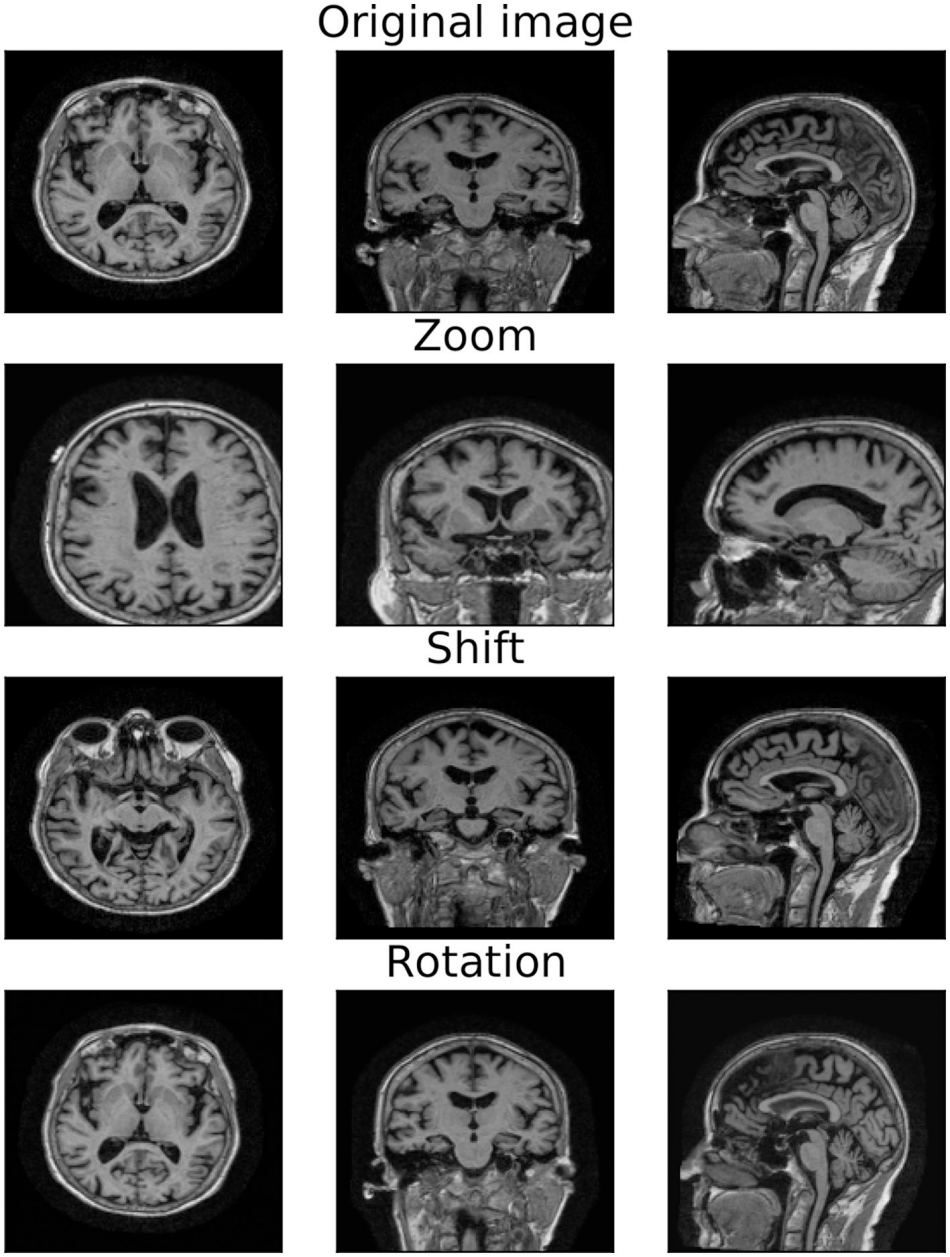
Original and transformed MRI image. 2D projections of the original MRI image **(first row)** and the augmented image obtained by applying *zoom* **(second row)**, *shift* **(third row)**, and *rotation* **(last row)** transformations.

Data augmentation is a common procedure that simultaneously addresses data scarcity and creates a model invariant to a given set of transformations (Shorten and Khoshgoftaar, 2019). Different augmentation strategies can result in varied training sets, affecting model performance and computational cost. In this study, the original set is augmented by applying, separately or simultaneously, *zoom, shift*, and *rotation* transformations, as shown in Figure 2 (see Supplementary Section 1.3 for details on the transformation parameters). To study the effect of different transformations and sample sizes on model performance, we compared the following three data augmentation strategies:

- **Strategy (A)**. To each image, we simultaneously apply all the transformations (i.e., a zoom by a random factor, a random shift, and a rotation by a random angle). The size of the augmented data will match the number of training samples *N*.
- **Strategy (B)**. To each image, we separately apply each transformation, generating three different distorted images. The size of the augmented data will be three times the number of training samples, 3*N*.
- **Strategy (C)**. To each image, we simultaneously apply all the transformations, as in strategy A. We repeat the process three times so that the number of augmented samples matches the one of strategy B (3*N*).

Therefore, strategies **(A)** and **(C)** rely on the same procedure, while strategies **(B)** and **(C)** generate the same number of samples. Although other augmentation techniques (e.g., color transformation, adding noise, and random erasing) may be beneficial, a comprehensive study of data augmentation is beyond the scope of this work. Instead, our goal is to investigate whether and how slight variations in data augmentation choices, often underestimated, impact model performance. In order to avoid data leakage (Wen et al., 2020), data augmentation is performed only on the training set after dataset split, leaving validation and testing sets at the original sample size.

#### 2.3.2 Data processing (D5)

As already noted, ADNI images were collected with different protocols and scanning systems, hence they are very heterogeneous in size, see Table 2. To enable the use of ML methods, it is necessary to select a common volume size. This choice, often left unexplained in literature, defines fundamental characteristics of the pipeline, such as the amount of information contained in the image and the input space dimension, on which model choice and computational burden depend.

**Table 2:** 1.5 T1-weighted MRI scans.

**MRI size**	**CN**	**AD**	**Total**
256 × 256 × 184	8	8	16
256 × 256 × 170	40	34	74
256 × 256 × 160	4	0	4
256 × 256 × 166	97	82	179
256 × 256 × 162	0	1	1
192 × 192 × 160	117	86	203
256 × 256 × 146	1	0	1
256 × 256 × 161	2	0	2
256 × 256 × 180	38	32	70

Number of CN and AD MRI scans grouped by size.

In our experiments, images are downsized to 96 × 96 × 73. The principle guiding this choice derives from computational issues. We first reduced the image dimension, rescaling the image by 50% along all dimensions, and we then resized images to match the smallest one. An alternative strategy may be zero-padding to match the biggest image, but this would increase memory requirements. Finally, intensity normalization was applied omitting the zero intensity voxels from the calculation of the mean. This procedure allows having homogeneous data with a fixed size. Note that we do not select any Region Of Interest (ROI) (Long et al., 2017) within the images. Although this setup challenges the classification task, it eliminates the typically laborious and time-consuming feature engineering process.

### 2.4 Experimental setup

#### 2.4.1 Guide to the model choice (M2)

Choosing the optimal DL model is not straightforward, as the vast numbers of network and training parameters makes a “brute-force” model selection approach unfeasible. Here, we illustrate the model choices made a priori based on the issues posed by the examined task.

##### 2.4.1.1 Type of data

Working with 3D images presents computational and memory challenges. As a solution, several studies in the literature adopt three 2D projections of the MRI. Nevertheless, this approach requires three separate models, leading to increased overall wall-clock time. Moreover, extracting features from the 2D projections may result in the loss of crucial volumetric information and a simplified representation of the studied phenomenon. In this work, we adopted a 3D CNN that directly extracts volumetric features.

##### 2.4.1.2 Limited amount of data

To overcome the limited dataset size, we implemented the following strategies aimed at controlling model complexity and preventing overfitting: data augmentation; adding an ℓ_2_ penalty; and limiting the number of filters per layer. The latter method resulted in a substantial parameter reduction across the network. For instance, in a 2-layer CNN with 3 × 3 × 3 filters, reducing the number of filters to 32 to 8 in the first layer and from 64 to 16 in the second layer (25% of the initial values) leads to a considerable reduction of 93% in the number of learnable parameters (from 56,256 to 3,696).

##### 2.4.1.3 Memory capacity

3D models usually require a huge amount of memory capacity, that depends both on the input dimension and the model size. To reduce the required memory: i) we re-scaled the images to halve the data dimension; ii) we used stochastic gradient descent with a batch size that balances the memory cost while retaining a representative subset; iii) we balanced the number of filters and the batch size to reduce the computational burden of the activation layer.

#### 2.4.2 Model details

We report experiments on the CN/AD binary classification. A preliminary analysis, performed on 1.5T MRI data with a standard training/validation/test split (75%/15%/10%), denoted a very high variance due to the limited sample size of the testing set. For this reason, to guarantee a correct assessment of model performance and stability, we set up a stratified-K-fold cross-validation loop. We set K = 7, from Fold 0 to Fold 6 (training/validation/test, with a proportion of 70%/15%/15%), that ensures having enough data for the learning phase (M4). All folds were fully balanced, except for Fold 6 which had an unbalanced ratio between AD and CN samples as the total amount of samples per class do not match exactly. We further tested our model on the external dataset of 3T MRI scans (M6). Note that this task is particularly challenging because: i) the evaluation is subject to the domain shift problem, and ii) the training MRI scans have half the resolution of the external MRI exams.

**Figure 3:**
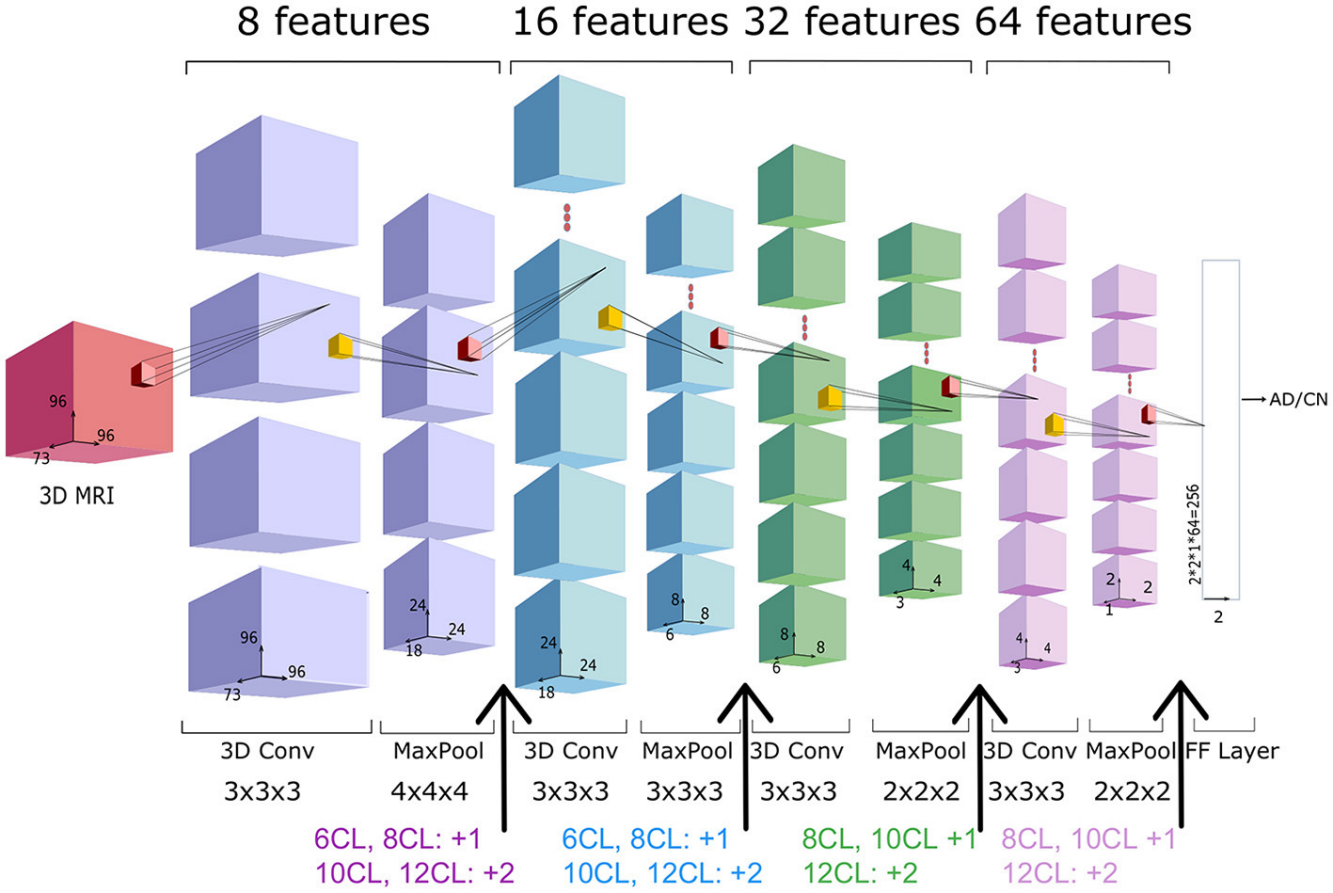
3D-CNN architecture. Architecture of the **4 CL** baseline network, composed by four blocks of a convolutional and pooling layers, followed by a fully connected (FC) layer. The total number of features (8*i) in the *i*-th convolutional layer is reported above each layer, whereas the filter dimension is reported below. In the experiments, we consider other four extended versions of the baseline architecture (6CL, 8CL, 10CL, 12CL) duplicating once or twice the convolutional layer preceding the arrows.

We adopted as baseline network an architecture with 4 Convolutional Layers (CL) followed by a fully-connected layer, as depicted in Figure 3. We will refer to this architecture as **4 CL** model. To investigate the optimal CNN depth, we inserted additional convolutional layers without pooling operations so that the number of layers is the only factor impacting in the model. Specifically, we added 2, 4, 6 and 8 convolutional layers in correspondence to the arrows of Figure 3. We refer to these models as **6 CL**, **8 CL**, **10 CL**, and **12 CL**. For instance, in the **10 CL** architecture 6 convolutional layers are added to the **4 CL** baseline: two layers are inserted in correspondence of the first and second arrows, and one layer in correspondence of the third and fourth arrows. Additional details on network and training parameters can be found in the Supplementary Section 2. In order to test model stability to initial random weights, each model was run 10 times (M4). Model selection was performed based on accuracy. The best one is further analyzed based on Confusion Matrix, Precision, Recall, F1-score, AUC and AUCPRC (M5).

All the experiments were conducted using Python version 3.8 and PyTorch 1.12.1, running on a Tesla K40c GPU. Samples identifiers and the Python code necessary to reproduce the experiments are available on GitHub (M1, M3).

## 3 Results

In the following, we compare 15 models obtained by combining different augmentation strategies with varying network depths, then we illustrate in detail the results of the best model. Results based on not-augmented data are not reported, as they were substantially worse than the ones obtained by using augmentation.

### 3.1 Architecture and augmentation choice

**Figure 4:**
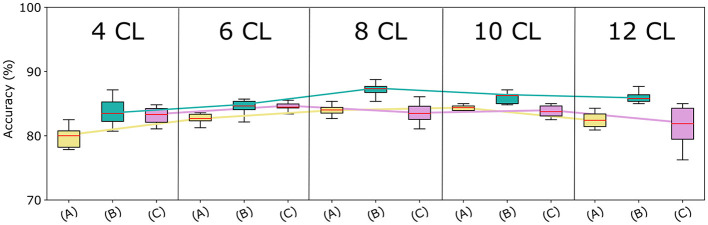
Models accuracy at varying of architecture depth and augmentation strategies. Comparison among the proposed CNN-based architectures with the three augmentation strategies, in terms of median accuracy on the validation set. The *y*-axis reports the model accuracy distribution on the 10 trials (%) and the *x*-axis presents varying augmentation strategies (A), (B), and (C) in 5 blocks—one for each CNN architecture.

We assessed the optimal architecture and augmentation strategy based on the accuracy on the validation set, which is shown in Figure 4. To verify the impact of these factors on the classification task, we performed a statistical analysis of the results obtained by the different models. Initially, we used the Shapiro-Wilk test (Shapiro and Wilk, 1965) to assess the normality of our data, which revealed that the data were not normally distributed. Consequently, we adopted a non-parametric approach to determine significant differences in models' performance. Specifically, we applied the Kruskal-Wallis test (Kruskal and Wallis, 1952) to compare performance across the 15 models. This analysis yielded a statistically significant difference (*p*-value = 7.45e-07), indicating that the classification task varies significantly among models with different augmentation strategies and network depth.

#### 3.1.1 Data augmentation

Strategy (A) (in yellow) considerably underperforms Strategy (B) (in green), regardless of the CNN architecture used. This can be attributed to the lower number of samples in the augmented data. Surprisingly, Strategies (A) and (C) (in fuchsia) achieve very similar accuracy for a higher number of layers. Finally, although Strategies (B) and (C) generate the same amount of data, Strategy (B) outperforms Strategy (C) across all network depths. To validate these findings, we repeated the Kruskal-Wallis test comparing models using strategy (A), (B), and (C), for each architecture. All tests resulted in p-values less than 0.05, confirming significant differences in performance across different augmentation strategies. Furthermore, as Strategy (B) resulted in the most effective data augmentation approach, we conducted additional statistical analysis on it. Specifically, we used the Conover-Iman test (Conover and Iman, 1979) for pairwise comparison between models based on strategy (B) and those employing different data augmentation strategies. Results revealed a significant difference between strategy (B) and strategy (A) for all network depth, and between strategy (B) and strategy (C) for the **8 CL**, **10 CL**, and **12 CL** architectures. These outcomes underscore the superiority of strategy (B) across all tested architectures, and demonstrate that applying affine transformations separately is more effective than applying them simultaneously.

#### 3.1.2 Network depth

**Figure 5:**
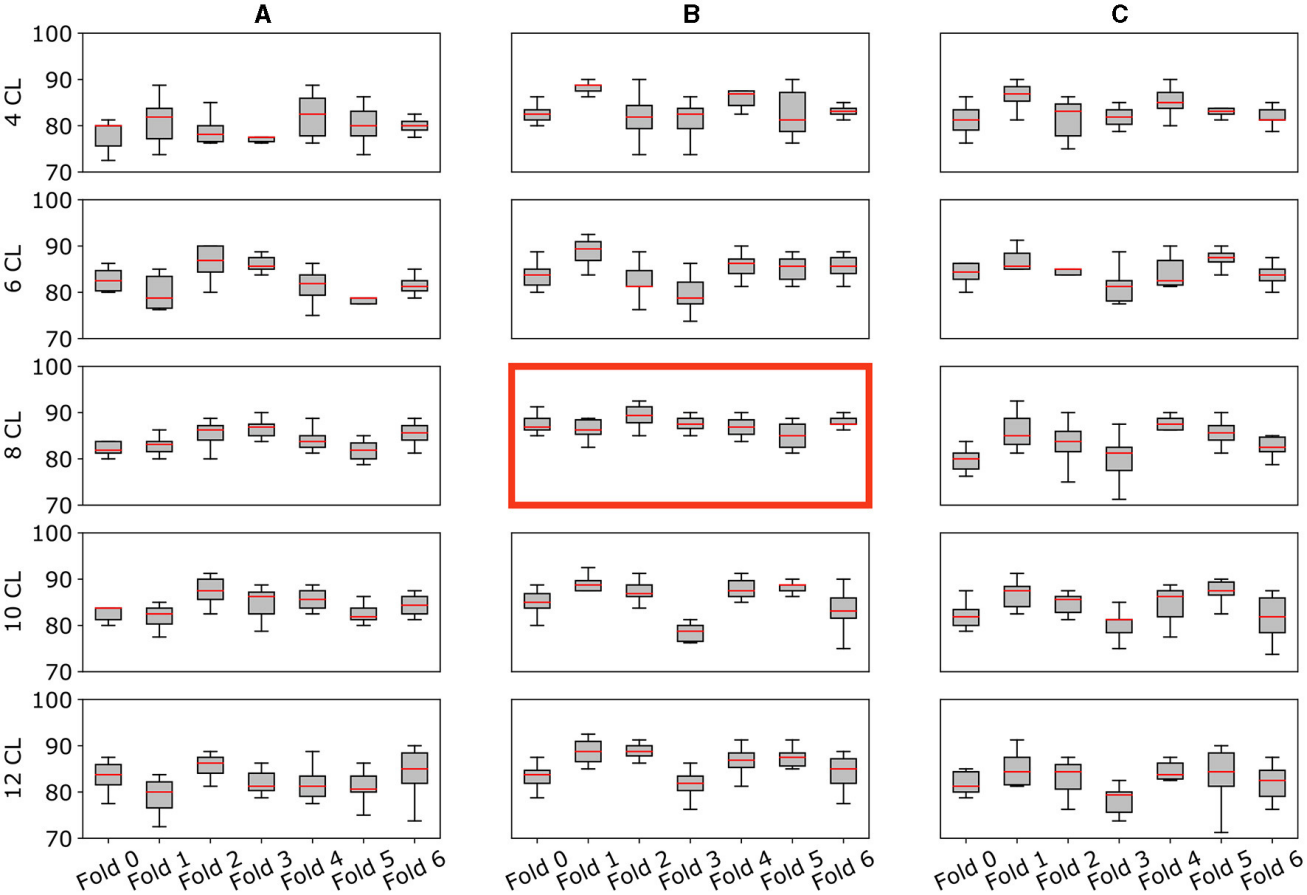
Model's performance and stability across folds. Multiple plots for the comparison of the validation accuracy for all architectures **(A–C)** and augmentation strategies (4CL, 6CL, 8CL, 10CL, 12CL). Each subplot reports the model accuracy on all 7-fold splits. Specifically, the y-axis reports the accuracy distribution on the 10 trials (%) for each fold (x-axis). The best model [8 CL, **(B)**] is highlighted with a red border.

The accuracy curves for all augmentation methods show a similar pattern: the best results are obtained for intermediate amounts of layers, while accuracy decreases for higher numbers of convolutional layers. The same behavior can be observed in Figure 5 where we report for each cross-validation fold the distribution of accuracy in the 10 trials. Using the Kruskal-Wallis test, we found that these differences across architectures were significant when using strategies (A) and (B).

The **8 CL** model with strategy (B) emerges as the best-performing combination, exhibiting greater stability within and across folds compared to the other combinations. Further details and specific results of the statistical analysis are available in the Supplementary material.

### 3.2 Best model performance and insight

The combination of a CNN with 8 convolutional layers and the (B) augmentation strategy [**8 CL**, (B)] turned out to be the best model, reaching an accuracy of 87.21 ± 0.88% on the validation set and 81.95 ± 1.26% on the testing set.

**Figure 6:**
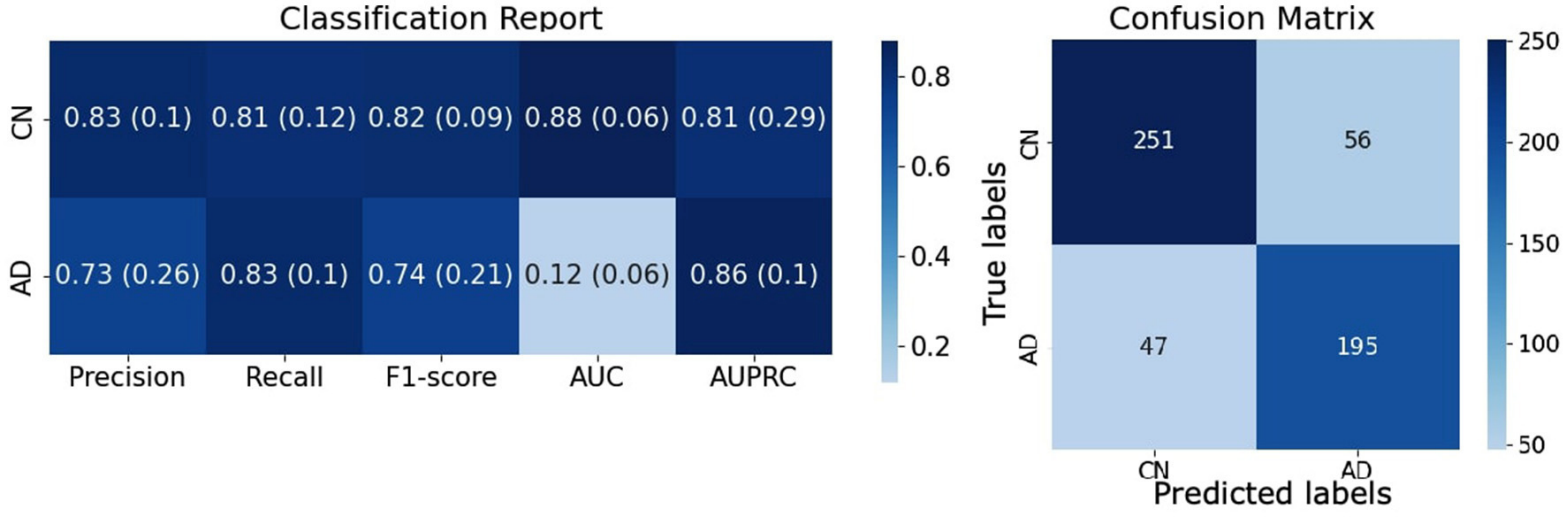
Evaluation of the [**8 CL**, (B)] model on the testing set. **(Left)** Complete evaluation of the model on CN and AD classes averaged over the 7 folds. **(Right)** Confusion matrix of the classification results counted over the 7 folds.

**Figure 7:**
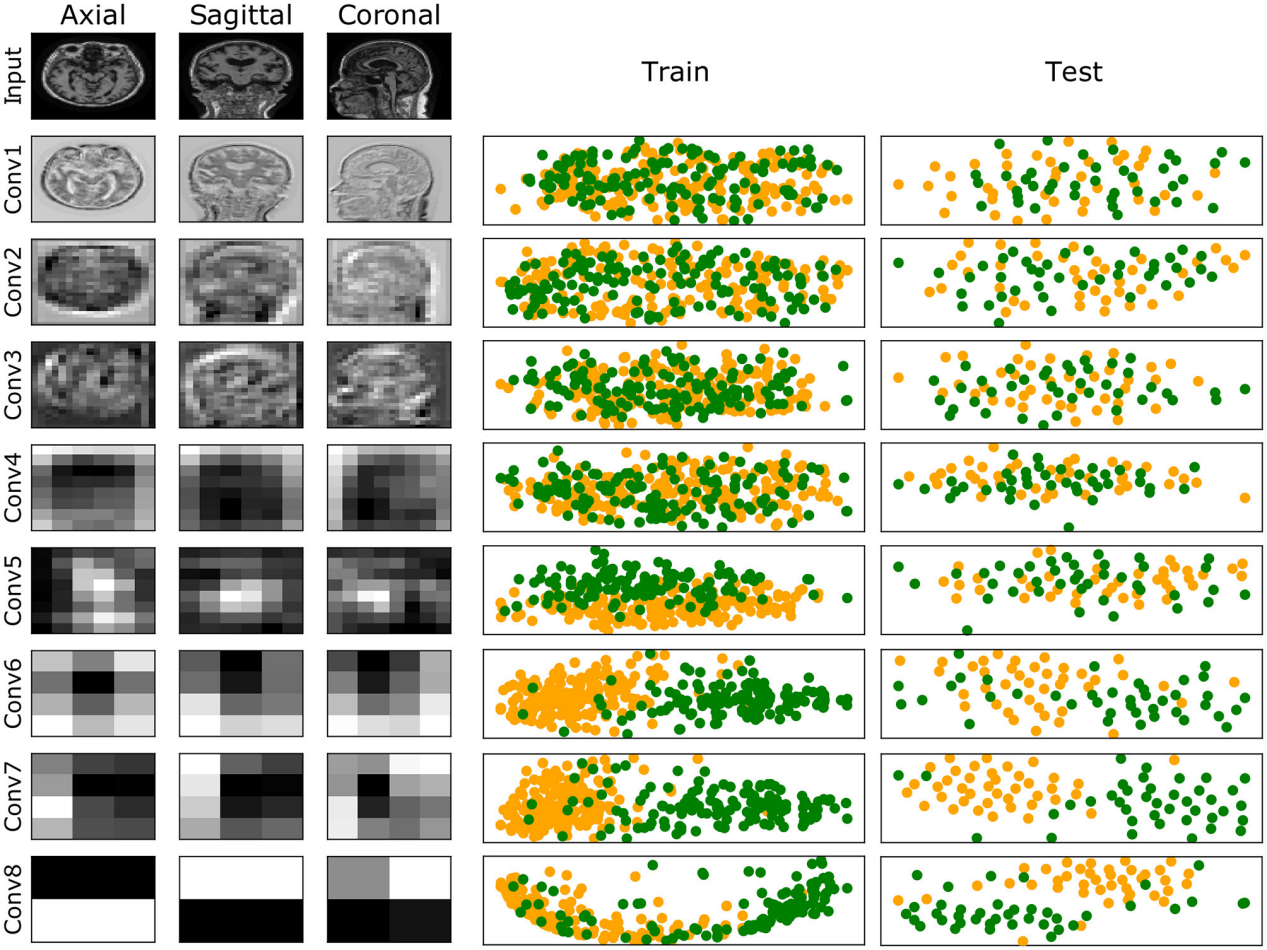
**(Left)** Illustration of the learned filters by the best model for one of the AD samples. Columns show filters for the three median planes, and rows show the filters for the input (raw data) and the convolutional layers at increasing depth. **(Right)** Training and test embeddings for each convolutional layer of the [**8 CL**, (B)] model projected by t-SNE. For increasing depth, AD (green) and CN (yellow) samples are better clustered.

A complete evaluation of this model is reported in Figure 6: left panel reports mean and standard deviation for Precision, Recall, F1-score, AUC and AUCPRC of CN and AD classes over the 7 folds; right panel shows the Confusion matrix obtained by counting True Positive, True Negative, False Positive, and False Negative scores over the 7 folds. Figure 7 gives an insight on the layers behavior and how they are learning the optimal model. The Left Panel displays the learned filters of every convolutional layer for one AD patient on the three considered median planes, i.e., *sagittal, coronal* and *axial*. It is clear that the filters capture more abstract features at increasing depth values. Panel (b) presents, for each convolutional layer, the layer outputs (*embeddings*) of training and test samples projected on a two-dimensional plane through t-distributed Stochastic Neighbor Embedding (t-SNE) (Van der Maaten and Hinton, 2008). Both projections show that the embeddings are more evidently clustered as the number of layers increases.

To further understand the properties and limits of the (**8 CL**, (B)) model, we assessed the effect of dropout, finding that it does not improve its performance (details in Supplementary Section 3.2). Also, we tested the model on an external dataset of 3T MRI scans, obtaining an accuracy of 71% and an AUC of 0.76 (a complete evaluation can be found in Supplementary Section 3.3).

## 4 Discussion

In this paper, we summarized a list of 5 items concerning *data handling* (D) and 6 items on *model design and assessment* (M), outlining the criteria that should be adhered to in order to ensure reliability, robustness, and reproducibility in ML for healthcare. Based on these criteria, we constructed an experimental pipeline for MRI-based binary classification of AD vs. CN subjects. Specifically, the experiments were conducted on a pre-processed subset of the ADNI dataset, consisting of 1.5T MRI scans collected during the screening ADNI1 phase (D1). This subset, previously pre-processed by ADNI experts, ensures high data quality (D2) and harmonization (D3). Although the dataset is balanced, its size is limited. To address potential overfitting and ensure reliable results, data augmentation (D4), model complexity reduction (M2), and resampling (M4) strategies were employed. All these aspects are thoroughly discussed (D5). The list of selected samples was made publicly available to enable benchmarking in further studies (M3), along with the Python code (M1).

Additionally, we thoroughly investigated the combined impact of data augmentation strategies (by varying the number of augmented data and the application of transformations) and architecture depth (M2), resulting in a total of 15 models. As reported in Section 2.2, these factors are often neglected in the literature, which typically aims to generate the largest possible number of augmented data and use state-of-the-art architectures (even when very large). Our findings demonstrate that improper settings for these experimental aspects can drastically hamper model performance, reducing accuracy by up to 10 points. Results showed that, independently of the adopted architecture, Strategy (B) always outperformed the others. As strategies (B) and (C) leverage the same amount of training samples, these results suggest that applying the affine transformations separately may help the model build invariance to each of them. Interestingly, strategies (A) and (C) show similar performances for intermediate-to-large models, even though strategy (A) relies on only one-third of the samples generated by strategy (C). We recall that Strategy (A) adopts the same combination of transformations as Strategy (B). This may indicate that the way transformations are combined and applied to the original data has a greater impact than the augmented dataset size itself. Future work will extend this investigation to other data augmentation strategies, including different types of transformation (e.g., color space transformations, Kernel filters, random erasing).

For all augmentation approaches, we found that the curve of the model accuracy at increasing depths tends to be a concave function, reaching the maximum for an intermediate depth value. Although the widespread notion for which deeper neural networks better generalize in a general framework, this result is in line with other studies (Zhang et al., 2021; Vento and Fanfarillo, 2019) in which authors showed that smaller models perform better when only a limited amount of data is available, as they are less subject to overfitting. Although we did not test them, this observation may extend to other SOTA architectures. Indeed, our **8 CL** CNN has 220k trainable parameters, while SOTA architectures are typically much larger. For example, ResNet18, ResNet50, and ResNet101 (He et al., 2016) consist of 11.7M, 25.6M, and 44.5M parameters, respectively. The smallest Vision Transformer model (ViT-Base) (Dosovitskiy et al., 2020) includes 86M parameters. EfficientNet-B1 (Tan and Le, 2019) and MobileNetV2 (Sandler et al., 2018), considered among the smallest SOTA architectures, have 7.8M and 3.5M parameters, respectively. Using larger SOTA models may be more effective when pre-trained to leverage transfer learning. However, it is important to note that the vast majority of pre-trained models have been trained on natural 2D images, and they are not immediately usable in the context of medical 3D scans. Future work will delve into these aspects.

The best model we identified is the combination of a CNN with 8 convolutional layers and the (B) augmentation strategy [**8 CL**, (B)]. The model accuracy in validation and testing is 87.21 ± 0.88% and 81.95 ± 1.26%, respectively, which is 4.2% increase in accuracy with respect to [**4 CL**, (B)] model. Also, Figure 5 shows how [**8 CL**, (B)] is more stable than all other models with respect to both cross-validation folds and training trials. These results appear in line with current SOTA studies relying on similar datasets. For instance, Pan et al. (2020) reach 84% of accuracy by using 499 1.5T MRI scans, and Xiao et al. (2017) obtain 85.7% using a dataset of 654 1.5T MRI images. Similarly to our work, Korolev et al. (2017) train a 3D-CNN model on 231 samples, showing 79% of accuracy. Nonetheless, we argue that a true comparison is not completely feasible as other works employ different datasets and data types, the number of samples varies both in training and testing sets, experimental designs are very heterogeneous and, most importantly, performance is always assessed on one trial, without any variability estimation. As an additional evaluation, we tested the best model in a *domain shift* context (M6), i.e., on 3T MRI data, reaching 71% of accuracy. We remark that this is a very challenging task as the image resolution deeply differs from the one in the training set.

To the best of our knowledge, this is the first work in the AD domain to delve into these modeling aspects and quantify their impact on performance estimation. Future work will extend this analysis to other architectures, different data augmentation transformations, and to a multi-class classification setting that includes MCI subjects.

## Data Availability

Publicly available datasets were analyzed in this study. This data can be found here: https://adni.loni.usc.edu/data-samples/access-data/.
