# Supplementary material for: Deep learning-based Alzheimer's disease detection: reproducibility and the effect of modeling choices
Source: Front Comput Neurosci. 2024 Sep 20;18:1360095. doi: 10.3389/fncom.2024.1360095 (PMC11451303; doi:10.3389/fncom.2024.1360095)
Supplement: Supplementary file 1 [file Data_Sheet_1.PDF]

Supplementary Material

1 DATA

1.1 The ADNI dataset

The ADNI cohort is a longitudinal multicenter study that aims at developing clinical, genetic and biomedical biomarkers for AD early detection. ADNI started collecting data since early 2000s and experienced four different phases, including over 2000 subjects affected by different degrees of cognitive impairment. However, different data modalities are not available for all subjects and therefore some scientific questions are still out of reach due to data scarcity.

In this work, we considered a pre-processed subset of 550 1.5T T1-weighted MRI scans from ADNI1. This includes 307 CN subjects and 243 AD patients.
Further, to evaluate the model ability to generalize to new datasets in the context of *domain shift*, we utilized 3T T1-weighted MRI scans as dataset for external validation. Specifically, this consists of 80 pre-processed images from ADNI1, including 47 healthy subjects and 33 patients with AD.

1.2 Data pre-processing

Experts by ADNI pre-processed the MRI exams in order to correct the image geometry distortion (by Gradwarp), the image intensity non-uniformity (by B1 calibration and N3). Finally, the images have been scaled for gradient drift using phantom data as detailed on the ADNI website. Note that not all these techniques have been simultaneously applied to each image, as the preprocessing procedure varies based on the acquisition system. It is worthwhile to mention that the ADNI screening folder contains images with a second type of scaling, referred to as *scaled_2*, which we used in place of the *scaled* when it is not available. The ADNI consortium did not report details on differences between the two scaling methods.

1.3 Data augmentation

In this work, we studied the effect of various augmentation strategies in which we differently combined three affine transformations, described in the following.

- *Zoom*. The in/out zoom is applied by randomly generating the zoom percentage, in a range from 0 to 20%.
- *Shift*. The shift is differently generated for each image dimension, so that shift< 0.4.
- *Rotation*. The rotation is defined by randomly generating a rotation angle, between -5 and 5 degrees, for each image dimension.

It is clear that there is not a golden rule for picking the optimal transformation parameters and that any value is somewhat arbitrary. Here we assume that brain image acquisitions differ one from another only for small variations. This assumption motivated our choices.

2 EXPERIMENTAL SETUP

We adopted as baseline network an architecture with 4 Convolutional Layers (CL) followed by a fully-connected layer (**4 CL** model). The number of filters in the i-th layer was set to $8*i$. All convolutional

Strategy	p-values
(A)	2.16e-08*
(B)	1.51e-05*
(C)	7.55e-02

Table S1. P-values resulting Kruskal-Wallis test comparing model performance across architectures, for each data augmentation strategy. P-values lower than 0.05 (*) indicates a statistical significant difference.

Architecture	p-values
**4CL**	2.96e-07*
**6CL**	9.13e-03*
**8CL**	1.86e-07*
**10CL**	3.01e-03*
**12CL**	7.45e-07*

Table S2. P-values resulting Kruskal-Wallis test comparing model performance across augmentation strategies, for each architecture. P-values lower than 0.05 (*) indicates a statistical significant difference.

layers have filters with a $3 \times 3 \times 3$ kernel. Padding is performed so that the original image and the feature map have the same size. We applied batch normalization to each convolutional layer. Successively, pooling was applied with decreasing size (i.e., $4 \times 4 \times 4$, $3 \times 3 \times 3$, $2 \times 2 \times 2$, $2 \times 2 \times 2$). The pooling size was chosen in order to decrease the layer size and, consequently, the computational cost. To investigate the optimal CNN depth, we inserted additional convolutional layers without pooling operations so that the number of layers is the only factor impacting in the model. Specifically, we 2, 4, 6 and 8 convolutional layers obtaining five models increasing number of layers 4, 6, 8, 10, 12.

Each model was trained using the Adam optimizer Kingma and Ba (2014) with a learning rate set to 0.001. We trained the network to minimize the cross-entropy loss function with ℓ_2-penalty weighted by 0.01. We allowed a maximum number of 200 epochs using early stopping if the performance does not increase after 20 epochs (patience). The batch size was 50. The choice of the described parameters was guided by the the criteria discussed in the Materials and Methods section and an exploratory analysis on the smallest model.

3 RESULTS

3.1 Architecture and augmentation choice

We first tested for normality using the Shapiro-Wilk test SHAPIRO and WILK (1965) and found that the data was not normally distributed. Consequently, we used a non-parametric test to determine if there were significant differences among model performances. Specifically, we employed the Kruskal-Wallis test Kruskal and Wallis (1952) to compare: 1) the performance of the 15 models; 2) the performance across architectures for each augmentation strategy; and 3) models with different augmentation strategies for each architecture. We found

- a statistically significant difference in performance across the 15 models, with a p-value of 7.45e-07;
- a significant difference of model performance across architectures for strategy (A) and (B), but not for strategy (C) (see Table S1);
- a significant difference of model performance across strategies, for each architecture (see Table S2).

Strategy	4CL	6CL	8CL	10CL	12CL
(A)	8.64e-07*	0.03*	4.13e-07*	0.01*	9.98e-07*
(C)	7.37e-01	0.61	2.47e-06*	0.01*	1.71e-05*

Table S3. P-values resulting from Conover's test comparing strategy (B) with the other data augmentation strategies, for each architecture. P-values lower than 0.05 (*) indicates a statistical significant difference.

As strategy (B) turned out to be the most effective one, we further investigated the obtained results by performing a pairwise comparison between models using strategy (B) and those employing different data augmentation strategies. Specifically, we adopted the Conover-Iman test Conover and Iman (1979). Table S3 reveals a significant difference between strategy (B) and strategy (A) for all architectures, and between strategy (B) and strategy (C) for the **8 CL**, **10 CL**, and **12 CL** architectures. These findings underscore the superiority of strategy (B) across all tested architectures and demonstrate that applying affine transformations separately is more effective than applying them simultaneously.

3.2 Best model performance and insight

Fig. S1 shows the model performance distribution on validation and testing set for all trials. Note that performance on Fold 6 of the testing set is associated with a very high variability due to the unbalanced AD/CN ratio, as anticipated in the Methods and Materials section. To better describe the behavior of the best model, we explored how some key characteristics change with evolving epochs, see the plots in Fig. S2.

In subplot 1, we can look at the cross-entropy loss function to assess proper model convergence as it decreases over time (epochs). In subplot 2, we can observe the training probability distribution of the predicted class, which is represented by the output of the last feed-forward layer. Here we verified that the learning process was not happening in an overfitting regime, as the training probability improves in terms of median and variability, but never reaching 100%. Subplots 3 and 4 display the training and validation accuracy across the epochs: their behavior is comparable, confirming that the model shows very good generalization properties. We noticed that both accuracy curves do not show a step-wise behavior. Hence, in further studies, one could reduce the patience (currently, 20) in the early stopping criterion and stop the training when the performance does not increase after a few epochs (e.g. 5 epochs). This would significantly reduce the computational cost and the training time of the experiments.

3.3 Ablation study on Dropout

Table S4 reports the classification accuracy on the validation and testing sets and the number of training epochs for the (**8 CL**, (B)) model in which dropout is applied with dropping probability ranging from 0 (i.e., no dropout) to 0.5. All the results are averaged on 10 trials. All models are comparable in terms of computational cost, whereas (**8 CL**, (B)) model without dropout slightly outperforms the others.

3.4 Generalization across image resolution

We evaluated the prediction ability of the (**8 CL**, (B)) model on unseen dataset that presents a shift domain. Specifically, we tested the generalization across image resolution. Results provided 71% of accuracy and an AUC curve of 0.76. Fig S3 reports the AUC curve (left) and the the confusion matrix (right).

REFERENCES

Conover, W. J. and Iman, R. L. (1979). *Multiple-comparisons procedures. Informal report*. Tech. rep., Los Alamos National Lab.(LANL), Los Alamos, NM (United States)

Kingma, D. P. and Ba, J. (2014). Adam: A method for stochastic optimization. *arXiv preprint arXiv:1412.6980*

Kruskal, W. H. and Wallis, W. A. (1952). Use of ranks in one-criterion variance analysis. *Journal of the American statistical Association* 47, 583–621

SHAPIRO, S. S. and WILK, M. B. (1965). An analysis of variance test for normality (complete samples). *Biometrika* 52, 591–611. doi:10.1093/biomet/52.3-4.591

Dropout	Validation accuracy	Testing accuracy	N. epochs
0	87.21 ± 0.88	81.95 ± 1.26	51
0.1	85.64 ± 2.56	80.21 ± 3.18	51
0.25	86.09 ± 1.34	80.57 ± 3.55	51
0.5	86.02 ± 1.69	80.96 ± 2.50	50

Table S4. Performance of (**8 CL**, (B)) model with and without dropout.

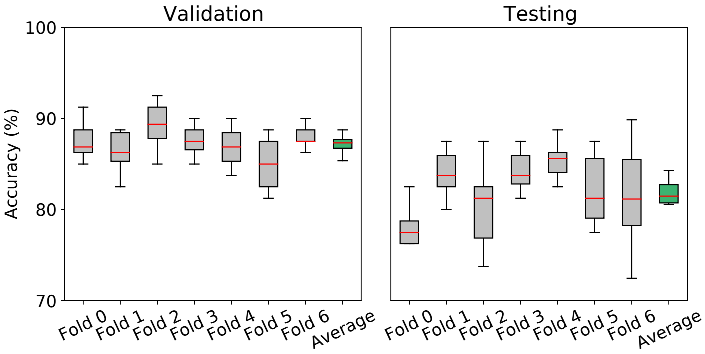

Figure S1. Validation and test accuracy of best model. Evaluation of (**8 CL**, (B)) model on validation and testing set in terms of percentage accuracy distribution on each fold (in silver) of the cross-validation and on average (in green), for all trials.

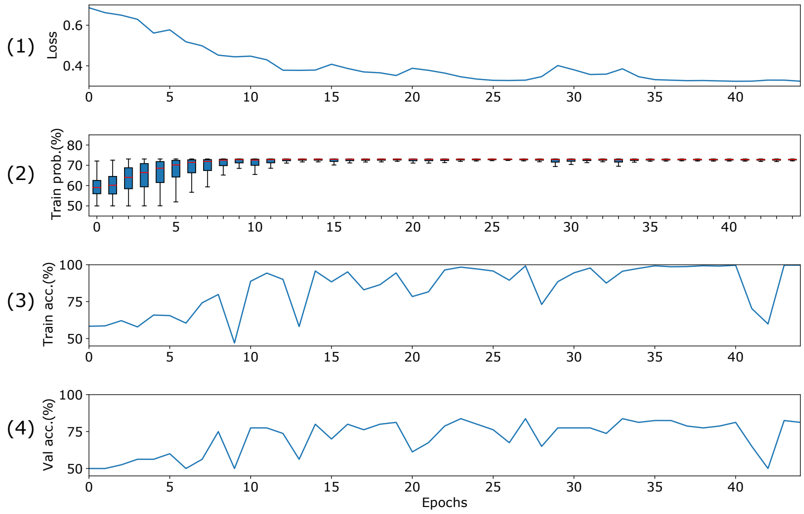

Figure S2. Evaluation of the best model during training. Four plots presenting, at evolving epochs, (1) the cross-entropy loss function values, (2) the probability distribution of training outputs, (3) training accuracy and (4) validation accuracy of the (**8 CL**, (B)) model.

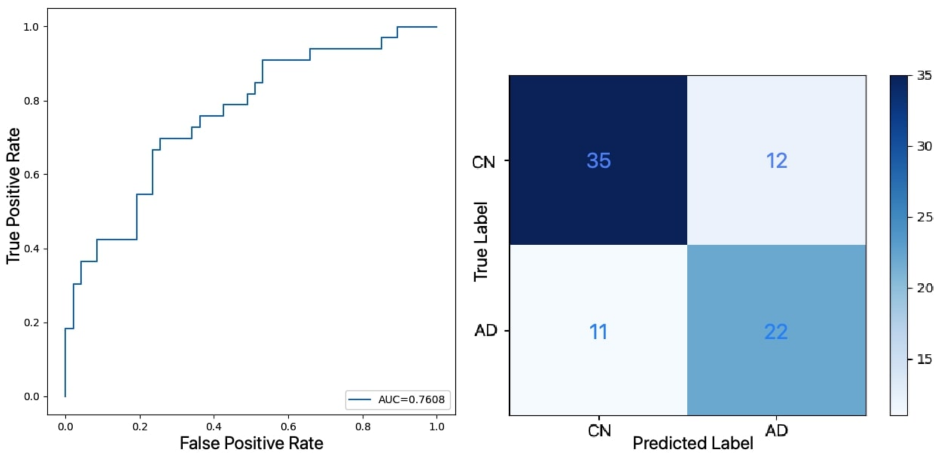

Figure S3. Results on 3T MRI scans. (left) AUC curve, (right) Confusion matrix.
